# Period2 is associated with immune cell infiltration and is a potential diagnostic and prognostic marker for hepatocellular carcinoma

**DOI:** 10.3389/fmolb.2023.1264553

**Published:** 2023-11-21

**Authors:** Xiaolin Chen, Qiaosu Zhao, Haibiao Wang, Kaijie Qiu, Xi Deng, Feng Xu

**Affiliations:** ^1^ Department of Gastroenterology, The Affiliated Lihuili Hospital of Ningbo University, Ningbo, China; ^2^ Department of Hepatobiliary Surgery, The Affiliated Lihuili Hospital of Ningbo University, Ningbo, China; ^3^ Department of Histopathology, Ningbo Clinical Pathology Diagnostic Center, Ningbo, China

**Keywords:** Period2, hepatocellular carcinoma, prognostic biomarker, tumor microenvironment, immune cell infiltration, Methylation

## Abstract

**Background**: Hepatocellular carcinoma (HCC) health challenge worldwide. Many studies showed that circadian rhythms play a critical role in tumor development. This study aimed to investigate the role of the circadian gene period2 (PER2) in HCC development and explore the possible mechanisms involved.

**Methods**: From fresh HCC tissues and paired paracancerous tissues, we measured PER2 mRNA and protein expression levels and calculated the correlations between PER2 expression and clinicopathological parameters in patients with HCC. We used transcriptome data from The Cancer Genome Atlas to mine the PER2 gene, including single gene difference analysis, single gene co-expression analysis, gene set enrichment analysis, immune infiltration analysis, and methylation analysis to explore its role and mechanism in HCC occurrence and development.

**Results**: PER2 expression levels were significantly lower in HCC tissues than in the paired paracancerous tissues. PER2 expression in HCC significantly correlated with neural invasion, Child-Pugh classification, and China liver cancer staging stage in HCC patients. The differentially expressed genes associated with PER2 were significantly enriched in mitochondrial oxidative phosphorylation, transcriptional translation, amino acid metabolism, and other related pathways. PER2 expression levels significantly correlated with immune cell infiltration and immune checkpoint genes and positively correlated with TP53 expression in HCC tissues. The DNA methylation status in eight CpG islands of the PER2 gene was associated with HCC outcomes.

**Conclusion**: PER2 is a potential diagnostic and prognostic biomarker and a promising therapeutic target in HCC.

## 1 Introduction

Liver cancer is one of the most common malignant digestive system tumors worldwide. The World Health Organization GLOBOCAN’s Global Cancer Statistics 2020 report stated that there were 19.3 million new cancer cases worldwide in 2020, of which liver cancer accounted for 4.7% of new cases (ranking sixth) and 9.9 million cancer deaths worldwide in 2020. Liver cancer accounted for 8.3% of deaths, ranking third (Sung et al., 2021). Hepatocellular carcinoma (HCC) is the predominant form of primary liver cancer, accounting for 75%–85% of primary liver cancers (Sung et al., 2021). For patients with HCC, despite progress in early diagnosis, chemotherapy, immunotherapy, and surgical treatment, outcomes remain unsatisfactory, with a 5-year survival rate of about 14.1% (Allemani et al., 2018). There is an urgent need to identify therapeutic targets and treatment options to improve survival (Hartke et al., 2017; El Jabbour et al., 2019; Xie et al., 2020).

Period2 (PER2) gene is essential to circadian rhythm maintenance in mammals (Shafi and Knudsen, 2019; Jiang et al., 2021). Disruption of circadian homeostasis is an independent risk factor for cancer (Shafi and Knudsen, 2019). Recent studies identified significant changes in PER2 expression in tumors such as chronic lymphocytic leukemia, kidney cancer, head and neck squamous cell carcinoma, and colorectal cancer (Momma et al., 2017; Xiong et al., 2018; Qiu et al., 2019; Wang et al., 2020). Low expression of PER2 protein in HCC tissues correlated with tumor diameter, portal invasion, TNM staging, and outcomes in HCC patients (Li et al., 2018).

The specific mechanism by which the circadian gene PER2 is involved in developing HCC is unknown. Therefore, we compared PER2 mRNA and protein expression in human HCC and paracancerous tissues and explored the relationship between PER2 expression and clinicopathological tumor characteristics. Bioinformatics analysis of the PER2 gene was performed using public databases to explore its potential functions and mechanisms of action in HCC development.

## 2 Materials and methods

### 2.1 Patients and databases

We collected HCC tissues and their paired adjacent tissues from 80 HCC patients who underwent radical HCC surgery at Ningbo Medical Center Lihuili Hospital from December 2021 to August 2022. Inclusion criteria were preoperative clinical diagnosis or pathological diagnosis of primary HCC and complete clinical data. Exclusion criteria were concurrent carcinomas *in situ* and absence of paracancerous tissues. All liver cancer tissues and their paired paracancerous tissues were collected during surgery. The specimens were partly snap-frozen in liquid nitrogen, stored at −80°C, and partly fixed in formalin and embedded in paraffin to create tissue wax blocks. The Ethics Committee of Ningbo Medical Center Lihuili Hospital approved the study (number: KY2021PJ231); patients provided informed written consent.

We downloaded the RNA sequencing (RNAseq) data in the Fragments Per Kilobase per Million (FPKM) format from the HCC Project from The Cancer Genome Atlas (TCGA) (https://portal.gdc.cancer.gov/). The RNAseq data in the FPKM format was converted into the transcripts per million reads format and log2-transformed. The Entrez IDs were converted to gene symbols using the “org.Hs.eg.db” (v3.10.0) R package.

### 2.2 Expression of PER2 mRNA and protein in HCC tissues and paired paracancerous tissues

Total RNA from tissues was extracted using a TransZol UP Plus RNA Kit (TransGen Biotech, Beijing, China), and the extracted total RNA (OD 260 nm/OD 280 nm > 1.8) was reverse transcribed into cDNA using a TransScript All-in-One First-Strand cDNA Synthesis Supermix for QPCR kit (TransGen Biotech, Beijing, China). Real-time fluorescence quantification was performed on an ABI 7500 PCR instrument with PerfectStart^®^ Green qPCR SuperMix (TransGen Biotech, Beijing, China). The following primers were PER2 forward, 5′-GAC​ATG​AGA​CCA​ACG​AAA​ACT​GC-3’; PER2 reverse, 5′-AGG​CTA​AAG​GTA​TCT​GGA​CTC​TG-3’; GAPDH forward, 5′- GCA​CCG​TCA​AGG​CTG​AGA​AC-3′, and GAPDH reverse, 5′-TGG​TGA​AGA​CGC​CAG​TGG​A-3’. The 2^−ΔΔCt^ method was used to compare the mRNA levels of liver cancer samples and their paired adjacent tissues.

Total protein was extracted as protein lysates, then protein quantification and sample preparation were performed. Protein samples were separated using 7.5% Express Cast polyacrylamide gel electrophoresis (NCM Biotech, Suzhou, China) and transferred to polyvinylidene fluoride membranes (Millipore Company, United States). The membranes were blocked with quick blocking buffer (NCM Biotech, Suzhou, China) at room temperature for 10 min, then incubated overnight at 4°C with a primary antibody working solution (PER2 Mouse monoclonal antibody, 67513-1-Ig, 1:5000, Proteintech Company, United States; β-tubulin rabbit monoclonal antibody, A12289, 1:1000, ABclonal, China). The next day, the membranes were washed three times with Tris-buffered saline-Tween was incubated with a secondary antibody working solution (HRP-conjugated Affinipure Goat Anti-Mouse IgG (H + L), SA00001-1, 1:2000, Proteintech Company, United States; HRP-conjugated Affinipure Goat Anti-Rabbit IgG (H + L), SA00001-2, 1:2000, Proteintech Company, United States) for 2 h at room temperature, then the membranes were washed three times with Tris-buffered saline-Tween. The chemiluminescence method was used to expose the membranes in Image Quant LAS 500 instrument. Finally, the gray value of protein bands was analyzed using ImageJ software, and protein expression levels in liver cancer samples and their paired adjacent tissues were compared.

The paraffin sections of specimens were stained with PER2 protein by Ningbo Pathological Center. Immunohistochemical staining score: In 400 times visual field, randomly select five visual fields, each counting 200 cells, and score the positive rate and intensity of staining. The PER2 staining positive rate score was obtained according to the proportion of positively stained cells. The score of the staining range was 0 (0%), 1 (1%–25%), 2 (26%–50%), 3 (51%–75%), and 4 (76%–100%). PER2 staining intensity score: negative 0, light yellow 1, light brown 2 and tan 3. The product of the positive staining rate score and staining intensity score was used to judge the positivity grade. Samples were classified as 0 negative (−), 1–4 weak positive (+), 5–8 moderate positive (++), or 9–12 strong positive (++). The average of five visual fields was recorded.

### 2.3 Correlation analysis between PER2 expression level and clinicopathological features of patients with HCC

According to the results of immunohistochemical staining of PER2 protein in liver cancer tissues, liver cancer tissues were divided into a low expression group where PER2 was negative (−) or weakly positive (+) and a high expression group where PER2 was moderately positive (++) or strongly positive (++). We recorded clinical data corresponding to liver cancer tissues, including gender, age, operation mode, smoking and drinking habits, body mass index (BMI), serum α-fetoprotein (AFP) level, whether they are infected with hepatitis B virus, tumor number, tumor maximum diameter, histological grade, microvascular invasion risk grading, Child-Pugh grading, China liver cancer staging (CNLC) to calculate correlations between PER2 expression and clinicopathological parameters and oncology behavior in patients with HCC.

### 2.4 Functional enrichment analysis of PER2-related differentially expressed genes (DEGs) in HCC

HCC patients were divided into high PER2 expression and low PER2 expression groups. DEGs between the two groups were analyzed using the “DESeq2” (V1.26.0) R package (Love et al., 2014), and the threshold parameters of differential analysis were set to |log2(FC)| >1.5 and p. adj <0.05. We used the “ggplot 2"(V3.3.3) R package to visualize DEGs in volcano and heat maps.

The “ClusterProfiler” (v3.14.3) R package (Yu et al., 2012) was used for the functional annotation and Gene Set Enrichment Analysis (GSEA) of the DEGs. The curated reference genesets from the MsigDB file (https://www.gsea-msigdb.org/gsea/msigdb/index.jsp): c2. cp.v7.2. symbols.gmt were selected for GSEA (Subramanian et al., 2005). The analysis results were significantly enriched with |NES| > 1, p. adj <0.05, and we used q-value <0.25 as thresholds.

### 2.5 Analysis of immune cell infiltration of PER2 in HCC

The ssGSEA algorithm in the “GSVA” (v1.34.0) R package (Hänzelmann et al., 2013) evaluated the tumor infiltration of 24 immune cell types in HCC tissues. These 24 immune cell markers were plasmacytoid dendritic cells (pDC), cytotoxic cells, dendritic cells (DC), T cells, B cells, CD56 bright natural killer cells (NDC), CD56 bright natural killer cells (NK CD56bright cells), gamma delta T cells (Tgd), immature dendritic cells (iDC), macrophages, effector memory T cells (Tem), regulatory T cells (TReg), type 1 helper cells (Th1 cells), neutrophils, CD56 dim natural killer cells (NK CD56dim cells), mast cells, T follicular helper cells (TFH), activated dendritic cells (aDC), type 2 helper cells (Th2 cells), CD8 T cells, natural killer cells (NK cells), eosinophils, Type 17 helper cells (Th17 cells), Central memory T cells (Tcm), and helper T cells (T helper cells) (Bindea et al., 2013). The immune infiltration in HCC tissues was assessed using the StromalScore, ImmuneScore, and ESTIMATEScore algorithms in the “estimate” (v1.0.13) R package. The relationship between PER2 expression, immune cell infiltration status, and immune cell markers was determined using Spearman correlation analysis.

### 2.6 Correlation analysis of PER2 expression level with other core circadian rhythm genes, immune checkpoint genes, and TP53 in HCC

Spearman correlation analysis determined the relationship between PER2 expression level and core circadian rhythm genes, immune checkpoint genes, and TP53. The core circadian rhythm gene (Jiang et al., 2021) and immune checkpoint gene (Hu et al., 2021) were obtained from a literature review. The correlation was considered significant, with *p* < 0.05 as the threshold. We used the “ggplot 2"(V3.3.3) R package to visualize the heat maps and scatter plots analysis results.

### 2.7 Analysis of DNA methylation status in the CpG islands of PER2

The DNA methylation status in the CpG sites of the PER2 gene was analyzed in TCGA using the MetSurv database (https://biit.cs.ut.ee/methsurv/). The prognostic value of the CpG methylation status of PER2 was evaluated in the HCC samples.

## 3 Results

### 3.1 The mRNA and protein expression levels of PER2 were significantly lower in HCC tissues than in their paired paracancerous tissues

**FIGURE 1:**
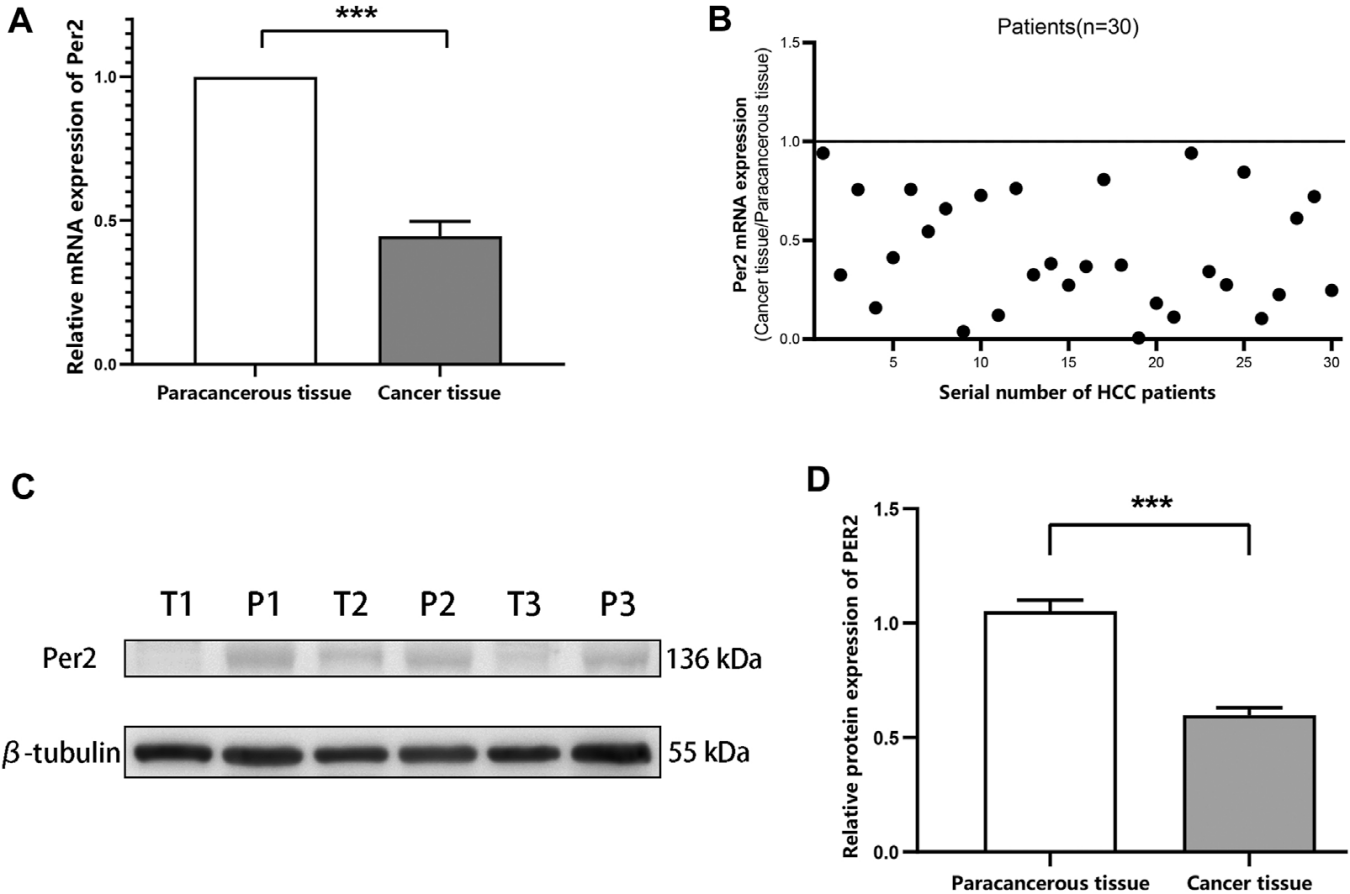
PER2 mRNA and protein expression levels in HCC tissues and paired paracancerous tissues. **(A,B)** The mRNA expression levels of PER2 were determined using qRT-PCR. **(C,D)** The protein expression levels of PER2 were determined using Western blotting. T, tumor tissue; P, paracancerous tissue; *p < 0.05, **p < 0.01, ***p < 0.001.

To measure mRNA and protein expression levels of the circadian gene PER2 in HCC tissues and paired paracancerous tissues, we randomly selected liver cancer tissues and paired paracancerous tissues from 30 liver cancer patients from our liver cancer sample library and measured PER2 mRNA expression levels using qRT-PCR (Figures 1A, B), randomly selected liver cancer tissues and paired paracancerous tissues from 54 liver cancer patients. We measured PER2 protein expression levels using Western blotting (Figures 1C, D). The mRNA and protein expression levels of PER2 in HCC tissues were significantly lower than in the paired paracancerous tissues (*p* < 0.001).

**FIGURE 2:**
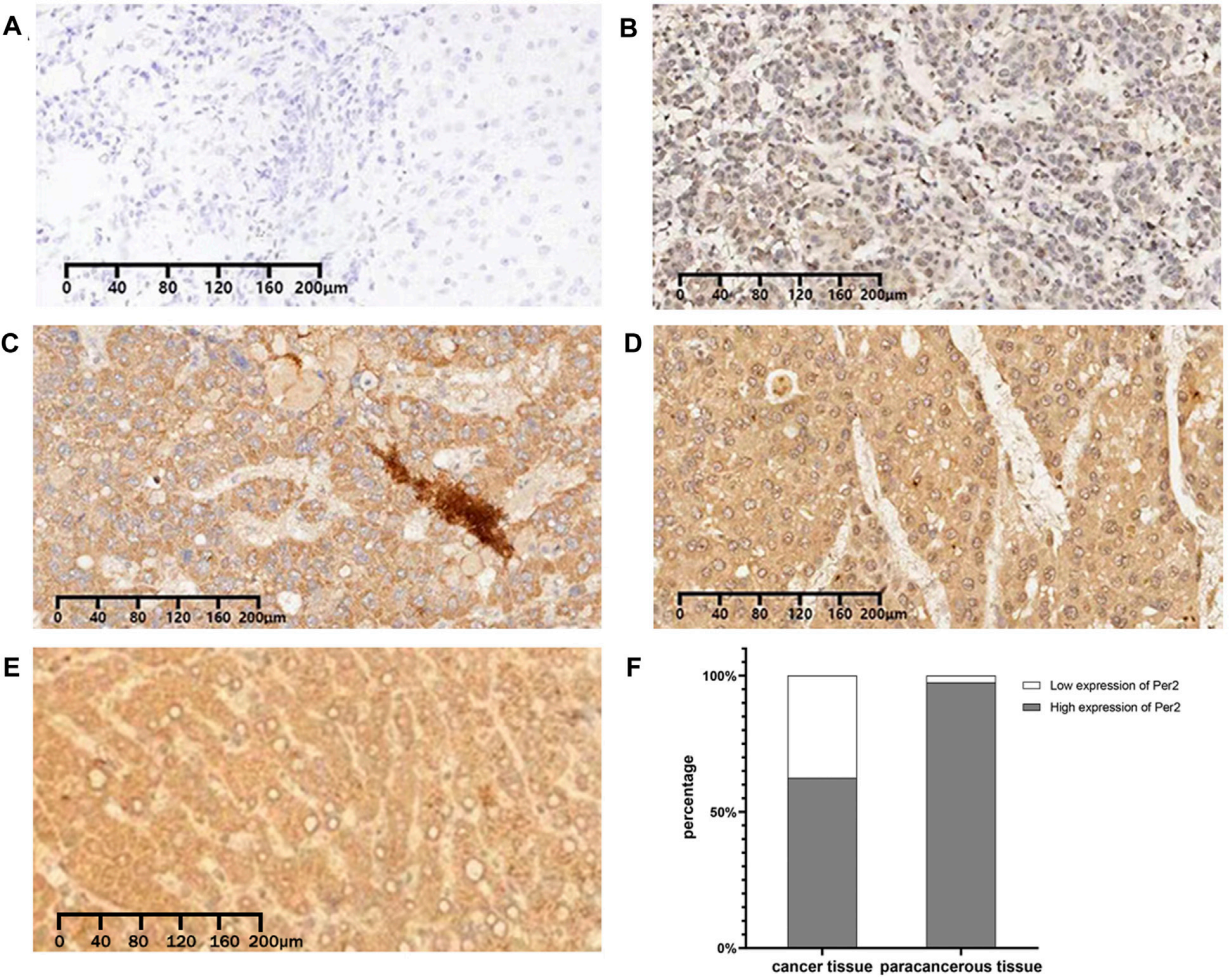
Immunohistochemical expression of PER2 protein in HCC tissues and paracancerous tissues. **(A–D)** The expression of PER2 was negative **(A)**, weakly positive **(B)**, moderately positive **(C)**, and strongly positive **(D)** in HCC. **(E)** The expression of PER2 was strongly positive in paracancerous tissues. **(F)** Stacked column diagram of PER2 protein expression in hepatocellular carcinoma and paracancerous tissues. IHC staining, ×100.

We measured the expression of PER2 protein in 80 pairs of HCC tissues and paired paracancerous tissues using immunohistochemical staining. The Ningbo Pathology Center performed paraffin tissue sections of the specimens and immunohistochemical staining scoring. A negative (−) or weakly positive (+) staining result indicated low PER2 expression; a moderately positive (++) or strongly positive (++++) staining result indicated high PER2 expression. In liver cancer tissues, high expression of PER2 protein accounted for 62.5%, and low expression of PER2 protein accounted for 37.5%. In paracancerous tissues, high expression of PER2 protein accounted for 97.5%, and low expression of PER2 protein accounted for 2.5% (Figure 2). There was significantly less expression of PER2 protein in liver cancer tissues than in paracancerous tissues (*p* < 0.05) (Table 1).

**TABLE 1:** Result of immunohistochemical staining of PER2 protein in HCC and paracancerous tissues.

Group	N	PER2 expression		χ^2^	*p*-value
		Low	High		
Cancer tissues	80	30	50	30.625	<0.05
Paracancerous tissues	80	2	78		

### 3.2 Correlation between the expression of the PER2 gene and clinicopathological parameters and oncological behavior of patients with HCC

To analyze the relationship between the circadian rhythm gene PER2 expression level in liver cancer tissues and the clinicopathologic and oncological characteristics, we collected clinical data from 80 patients with liver cancer corresponding to liver cancer tissues. We recorded sex, age, operation mode, smoking and drinking habits, BMI, serum AFP level, whether they were infected with hepatitis B virus, tumor number, maximum tumor diameter, histological grading, microvascular invasion risk grading, Child-Pugh grading, and CNLC staging. Samples were divided into high and low PER2 expression groups, with 50 people in the high-expression group and 30 in the low-expression group (Table 2). PER2 expression significantly correlated with nerve invasion (*p* = 0.017), Child-Pugh grading (*p* = 0.004), and CNLC staging (*p* = 0.004).

**Table 2:** Clinicopathological characteristics of HCC patients with high- and low-PER2 expression levels.

Characteristics	Number	PER2 expression levels		*p*
		Low	High	
Total number of patients	80	30	50	
Gender				0.306
Male	70	28	42	
Female	10	2	8	
Age				0.649
<50	14	6	8	
≥50	66	24	42	
Operation				0.645
Hepatectomy	75	29	46	
Liver transplantation	5	1	4	
Drinking				0.712
No	54	21	33	
Yes	26	9	17	
Smoking				0.902
No	54	20	34	
Yes	26	10	16	
BMI				0.229
<18 kg/m^2^	5	0	5	
18∼24 kg/m^2^	39	15	24	
≥24 kg/m^2^	36	15	21	
AFP				0.424
<200 μg/L	60	21	39	
≥200 μg/L	20	9	11	
HBV				0.083
No	16	3	13	
Yes	64	27	37	
Tumor number				0.144
≤3	72	25	47	
>3	8	5	3	
Vascular tumor thrombus				0.628
No	76	28	48	
Yes	4	2	2	
Maximum tumor diameter				0.649
<5cm	66	24	42	
≥5cm	14	6	8	
Histological grade				0.124
Poor differentiation	30	15	15	
Moderate differentiation	41	11	30	
High differentiation	9	4	5	
Satellite lesions				0.706
(-)	73	28	45	
(+)	7	2	5	
MVI risk grade				0.383
M0	45	15	30	
M1 + M2	35	15	20	
Nerve invasion				0.017
(-)	76	26	50	
(+)	4	4	0	
Hepatic capsule invasion				1.000
(-)	70	26	44	
(+)	10	4	6	
Involve peripheral organs				0.553
(-)	77	28	49	
(+)	3	2	1	
Child-Pugh grade				0.004
A	72	23	49	
B	8	7	1	
CNLC stage				0.004
Ⅰ	62	18	44	
Ⅱ+Ⅲ+Ⅳ	18	12	6	

### 3.3 DEGs with high and low PER2 expression in HCC

**FIGURE 3:**
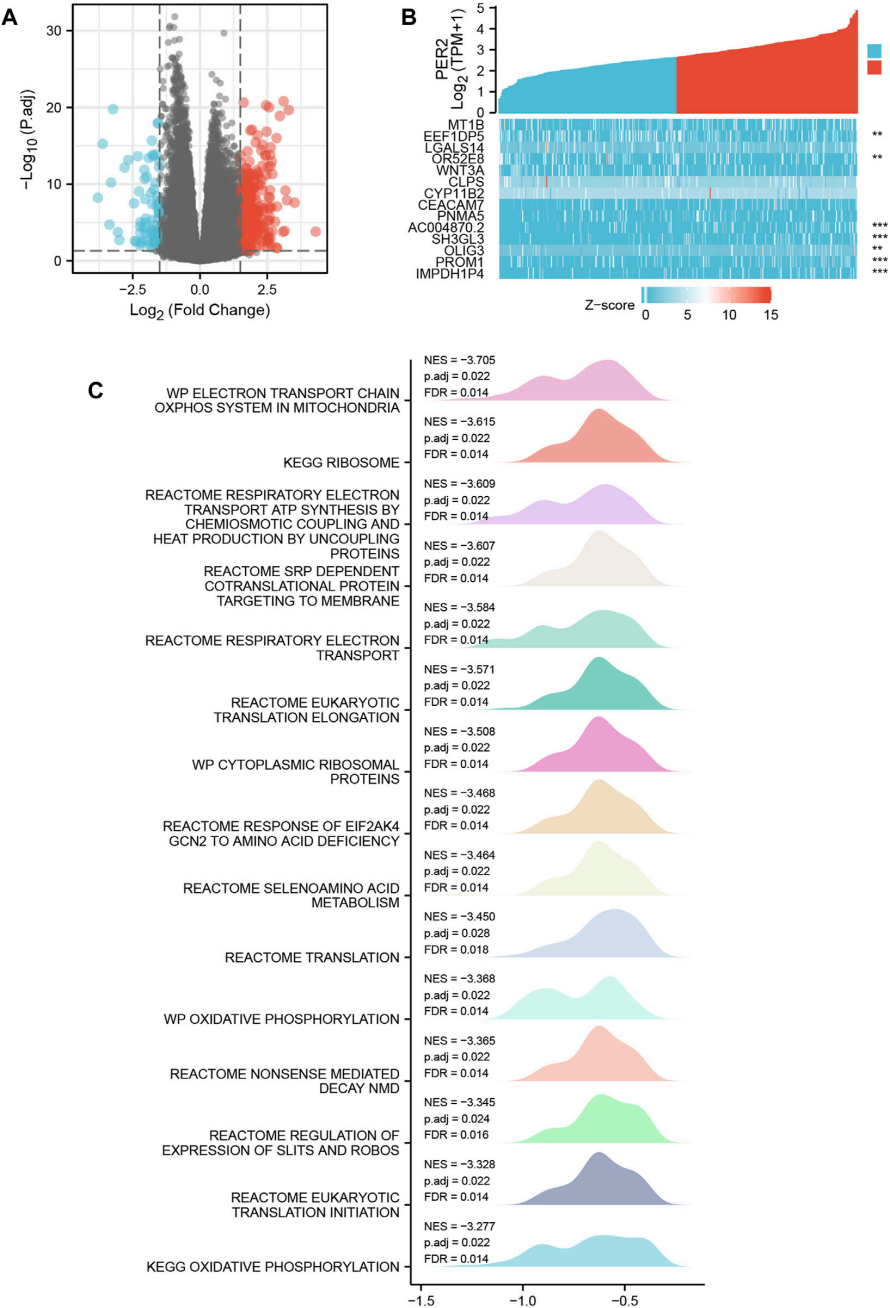
PER2-related differentially expressed genes (DEGs) and their functional enrichment analysis in HCC. **(A)** The volcano plot and **(B)** the correlation heat map of DEGs with high and low expression of PER2 in HCC. *p < 0.05, **p < 0.01, ***p < 0.001. **(C)** Gene Set Enrichment Analysis of PER2 expression-related DEGs in HCC.

Based on the transcriptome data of HCC in TCGA, 424 HCC patients were divided into two groups according to the median expression value of the PER2 gene, and the differential expression between the two groups was analyzed. Taking |log2(FC)| >1.5 and p. adj <0.05 as threshold parameters, DEGs related to PER2 expression were obtained. Compared with the low expression group, there were 365 DEGs in the high PER2 expression group, of which 295 were upregulated, and 70 were downregulated (Figure 3A). Fourteen crucial DEGs were presented as a co-expression heat map (Figure 3B).

### 3.4 Functional enrichment analysis of PER2-related DEGs in HCC

To study the biological function and signal pathways related to the PER2 gene in HCC, we used the transcriptome data from TCGA to group samples according to the median expression value of PER2 and obtained the DEGs related to PER2. We used the “Cluster Profiler” R package and the GSEA of PER2-related DEGs in HCC patients; the possible functions or pathways involved in PER2 gene expression were inferred. The predefined gene set used in GSEA came from the c2. cp.v7.2. symbols.gmt gene set in the MSigDB database. The PER2-related DEGs were enriched in mitochondrial oxidative phosphorylation, transcription and translation, amino acid metabolism, and other related pathways (|NES| > 1, p. adj <0.05, q value < 0.2) (Figure 3C).

### 3.5 The expression of PER2 in HCC is related to immune cell infiltration

**FIGURE 4:**
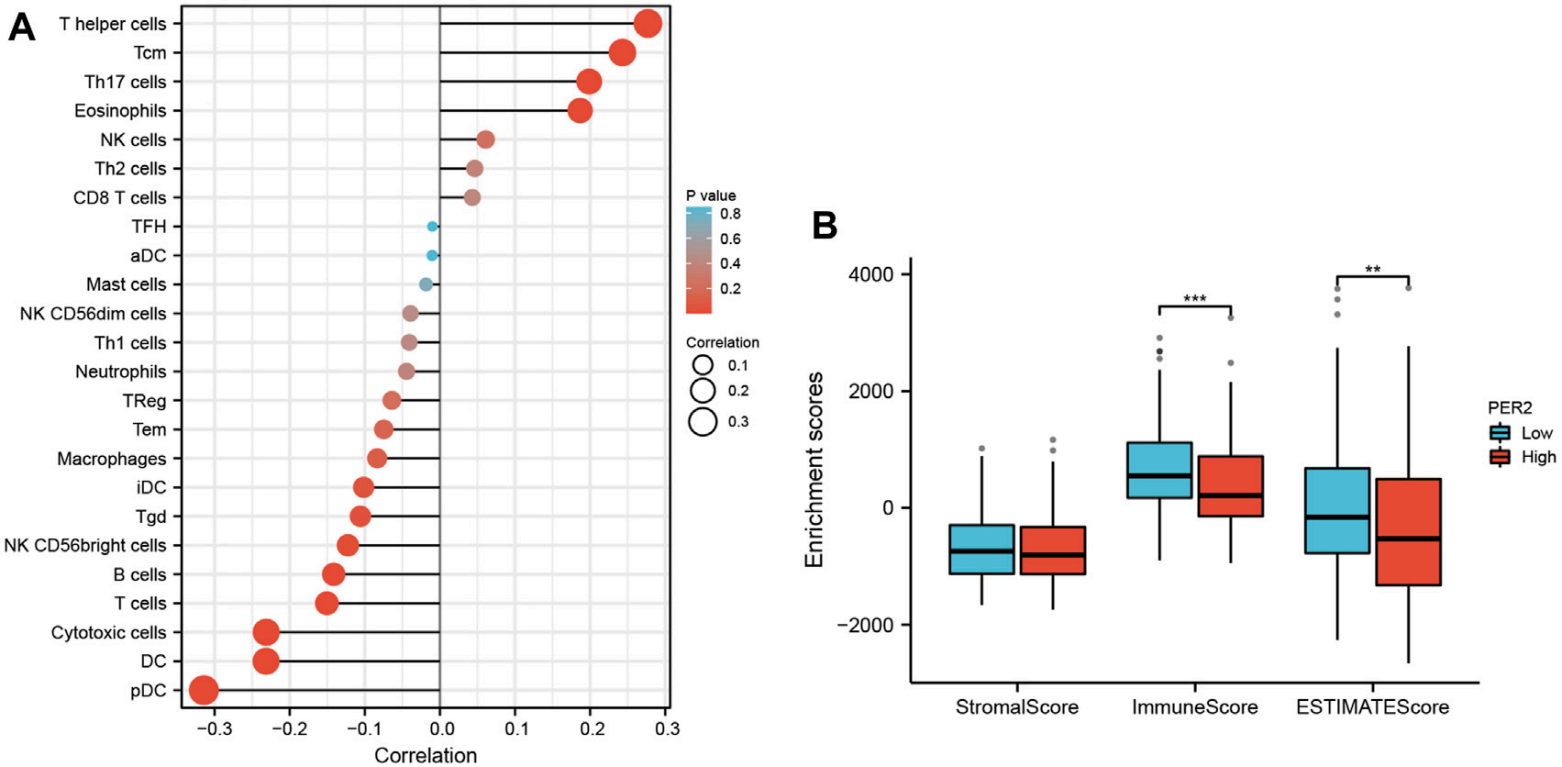
The correlation analysis between immune cell infiltration and PER2 expression in HCC. **(A)** Spearman correlation analysis results between infiltration levels of 24 immune cell types and PER2 expression levels in the HCC tissues. **(B)** Spearman correlation analysis results between the immune infiltration score and PER2 expression levels in the HCC tissues. *p < 0.05, **p < 0.01, ***p < 0.001.

Transcriptome data from HCC in TCGA were used to evaluate the infiltration status of 24 immune cell types using the ssGSEA algorithm in the “GSVA” R package. The correlation between PER2 expression and immune cell infiltration was calculated using Spearman correlation analysis. The expression of PER2 mRNA was correlated with many immune cells, including plasma cell-like pDC, cytotoxic cells, DC, T cells, B cells, NK CD56 bright cells, Tgd, iDC, eosinophils, Th17 cells, Tcm, and helper cells (Figure 4A; Table 3). We also used the “ESTIMATE” Score package to score the immune infiltration of HCC tissues. The results showed that the low expression of PER2 was significantly correlated with the immune infiltration score (*p* < 0.001) and with the immune and matrix comprehensive score (*p* < 0.01) (see Figure 4B). PER2 expression in HCC is related to immune cell infiltration.

**TABLE 3:** Correlation analysis results between infiltration levels of 24 immune cell types and PER2 expression levels in HCC tissues.

Immune cell	Correlation	*p*
pDC	−0.309	<0.001
Cytotoxic cells	−0.237	<0.001
DC	−0.232	<0.001
T cells	−0.158	0.002
B cells	−0.151	0.004
NK CD56bright cells	−0.132	0.011
Tgd	−0.112	0.030
iDC	−0.111	0.031
Macrophages	−0.093	0.074
Tem	−0.078	0.13
TReg	−0.074	0.154
Th1 cells	−0.051	0.326
Neutrophils	−0.049	0.348
NK CD56dim cells	−0.046	0.376
Mast cells	−0.028	0.588
TFH	−0.02	0.697
aDC	−0.016	0.752
Th2 cells	0.044	0.396
CD8 T cells	0.046	0.373
NK cells	0.061	0.238
Eosinophils	0.177	<0.001
Th17 cells	0.204	<0.001
Tcm	0.253	<0.001
T helper cells	0.271	<0.001

### 3.6 PER2 expression in HCC is related to the expression of many core circadian rhythm genes, immune checkpoint genes, and TP53

**FIGURE 5:**
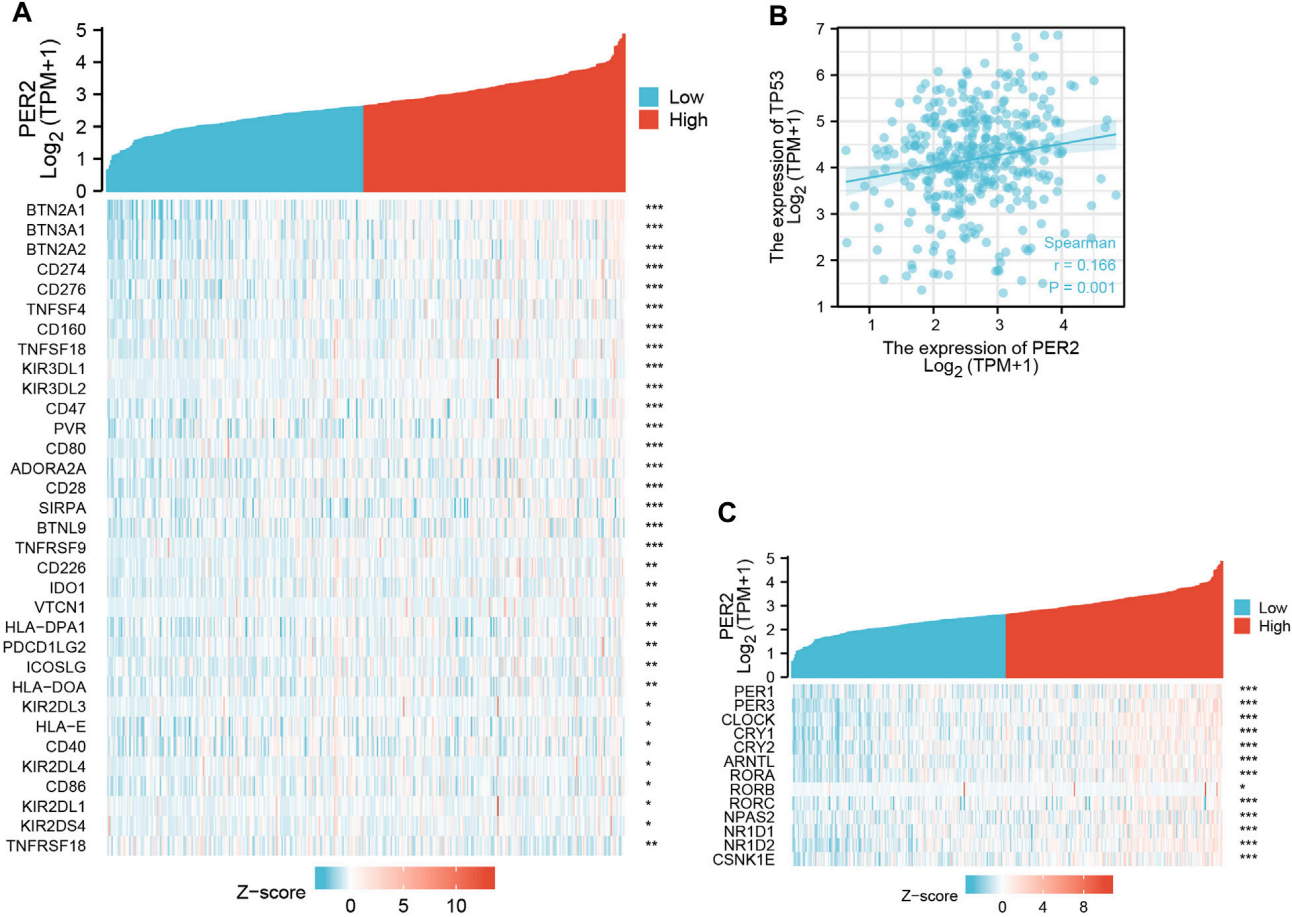
The correlation analysis between PER2 expression and **(A)** immune checkpoint genes, **(B)** TP53, and **(C)** other core circadian rhythm genes in HCC. *p < 0.05, **p < 0.01, ***p < 0.001.

A literature review revealed that there are a least 15 core circadian rhythm genes: PER1, PER2, PER3, CLOCK, CRY1, CRY2, ARNTL/BMAL1, TIMLESS/TIM, RORA, RORB, RORC, NPAS2, NR1D1, NR1D2, and CSNK1E/CKIε (Jiang et al., 2021). Using the transcriptome data of HCC in TCGA, the correlation between the PER2 gene and other core circadian rhythm genes was analyzed and expressed as a thermogram (Figure 5C). PER2 expression correlated with many core circadian rhythm genes (*p* < 0.05), including PER3, CLOCK, and NR1D2 (correlation >0.5) (Table 4).

**TABLE 4:** The correlation analysis between PER2 and other core circadian rhythm genes expression.

Core circadian rhythm genes	Correlation	*p*
CLOCK	0.566	<0.001
PER3	0.558	<0.001
NR1D2	0.543	<0.001
CRY1	0.466	<0.001
CRY2	0.452	<0.001
RORA	0.421	<0.001
ARNTL	0.398	<0.001
RORC	0.39	<0.001
NR1D1	0.343	<0.001
CSNK1E	0.331	<0.001
PER1	0.315	<0.001
NPAS2	0.241	<0.001
RORB	0.13	0.012

Given the broad application of immune checkpoint inhibitors in anti-tumor treatment, we studied the relationship between PER2 expression and immunosuppression checkpoints. A literature review revealed 79 immune checkpoint genes (Hu et al., 2021). We analyzed the transcriptome data of HCC in TCGA to calculate the correlation between PER2 and immune checkpoint gene expression. PER2 positively correlated with 32 immune checkpoint genes and negatively correlated with one immune checkpoint gene (*p* < 0.05). These correlated immune checkpoint genes were presented as heat maps (Figure 5A).

TP53 is a tumor suppressor gene with low expression in normal cells and high expression in malignant tumors (Marei et al., 2021). Using correlation analysis of transcriptome data of HCC in TCGA, we found that PER2 expression positively correlated with TP53 expression (Figure 5B).

### 3.7 Correlation between methylation status of the PER2 gene and outcomes in HCC patients

**FIGURE 6:**
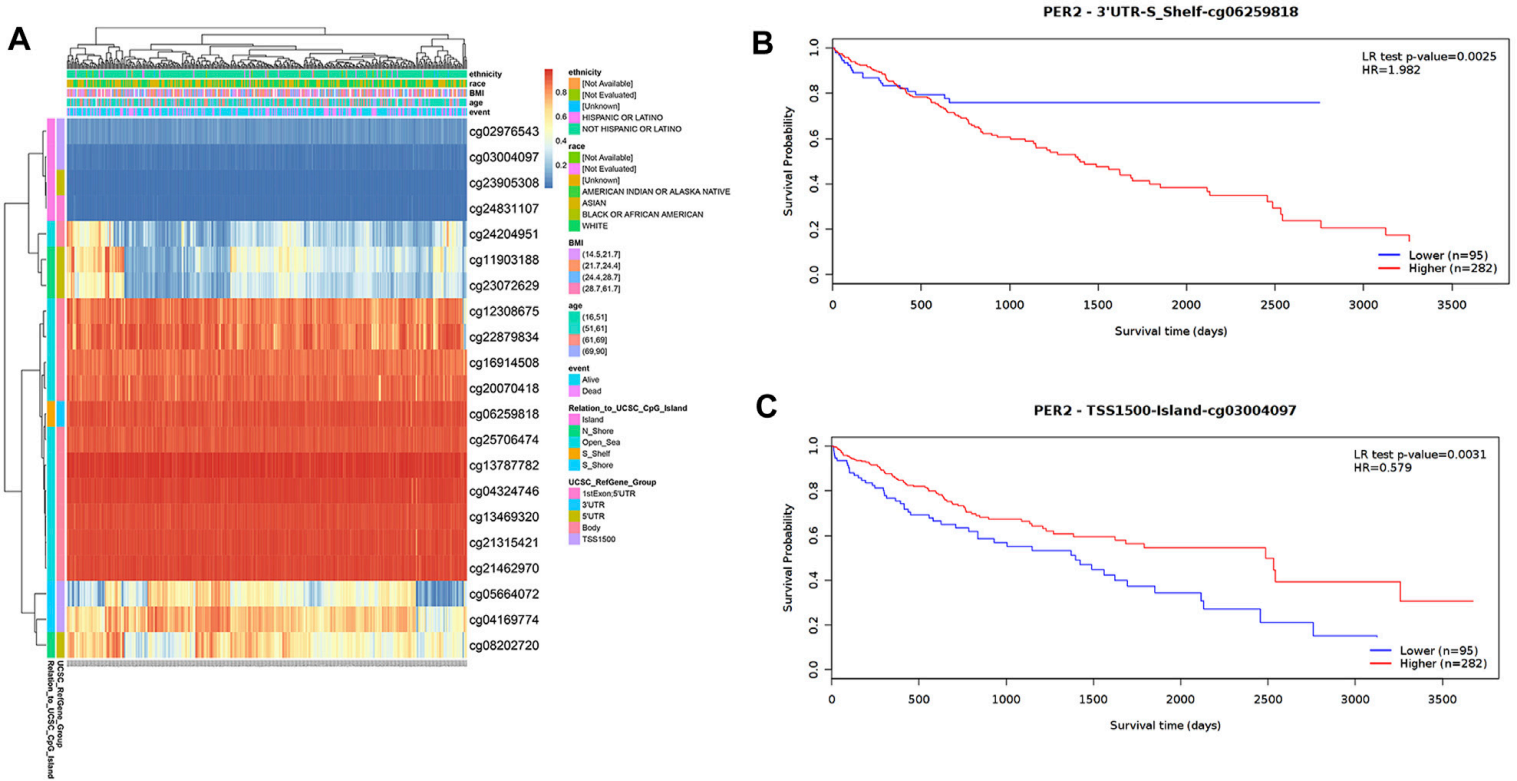
Analysis of DNA methylation status in the CpG islands of the PER2. **(A)** The visualization of 21 CpG sites on PER2 with methylation level in HCC. **(B,C)** The Kaplan-Meier survival curves of the DNA methylation of PER2 at cg06259818 **(B)** and cg03004097 **(C)** probes.

The DNA methylation level in the PER2 gene and the prognostic value of each CpG locus in the PER2 gene were analyzed using the MetSurv tool. The thermogram results revealed the methylation levels of 21 CpG sites in HCC (Figure 6A). The methylation levels of eight CpG sites (cg03004097, cg04169774, cg20070418, cg22879834, cg21315421, cg24831107, cg12308675 and cg06259818) were significantly correlated HCC outcomes (*p* < 0.05) (Table 5). Patients with hypermethylation of cg03004097 had better survival than patients with hypomethylation (*p* < 0.01, hazard ratio [HR] = 0.579), while patients with hypermethylation of cg06259818 had significantly worse survival outcomes than those with hypomethylation (*p* < 0.01, HR = 1.982) (Figures 6B, C). These findings suggest that the methylation status of the PER2 gene is related to HCC outcomes.

**TABLE 5:** Effects of methylation levels in the CpG sites of the PER2 gene on HCC outcomes.

CpG island	HR	*p*
TSS1500—Island—cg03004097	0.579	0.0024
TSS1500—S_Shore—cg04169774	0.703	0.044
TSS1500 - S_Shore - cg05664072	0.741	0.089
Body—Open_Sea—cg13787782	0.769	0.18
Body—Open_Sea—cg21462970	0.842	0.33
5′UTR—N_Shore—cg23072629	0.842	0.33
Body—Open_Sea—cg16914508	0.87	0.43
5′UTR—N_Shore—cg11903188	0.878	0.46
5′UTR—N_Shore—cg08202720	0.881	0.51
Body—Open_Sea—cg25706474	0.882	0.52
TSS1500—Island—cg02976543	1.134	0.48
Body—Open_Sea—cg13469320	1.229	0.24
Body—Open_Sea—cg24204951	1.29	0.23
5′UTR—Island—cg23905308	1.291	0.15
Body—Open_Sea—cg20070418	1.438	0.049
Body—Open_Sea—cg22879834	1.481	0.025
Body—Open_Sea—cg04324746	1.49	0.079
Body—Open_Sea—cg21315421	1.529	0.018
1stExon; 5′UTR—Island—cg24831107	1.562	0.038
Body—Open_Sea—cg12308675	1.645	0.024
3′UTR—S_Shelf—cg06259818	1.982	0.005

## 4 Discussion

Downregulation of PER2 expression is related to poor outcomes in several tumors, including chronic lymphocytic leukemia, renal cancer, head, and neck squamous cell carcinoma, colorectal cancer, and others (Momma et al., 2017; Xiong et al., 2018; Qiu et al., 2019; Wang et al., 2020). Previous studies showed that PER2 mRNA and protein in HCC tissue were lower than in paired adjacent tissues (Yang et al., 2014; Li et al., 2017; Li et al., 2018). These findings are consistent with the results of the present study. Based on the clinical and pathological data analysis of patients with liver cancer, PER2 protein expression in liver cancer tissue was significantly related to nerve invasion, Child-Pugh grading, and CNLC staging. These findings suggest that the circadian rhythm gene PER2 is critical in HCC.

Circadian rhythm regulation involves a large and complex interactive regulation network that maintains an organism’s steady state (Momma et al., 2017). Chronic disturbance of circadian rhythm is related to metabolic diseases, and is closely related to increased cancer risk (Crespo et al., 2021). Some core circadian rhythm genomes have become feedback loops of gene transcription and translation (Shafi and Knudsen, 2019). Basic helix-loop-helix heterodimeric transcription factors (CLOCK/BMAL1 or BMAL1/NPAS2) transcribe-translate the feedback loop in a negative feedback way, thus regulating the expression of CRY1, CRY2, PER1, PER2, and PER3 (Shafi and Knudsen, 2019). It is worth noting that circadian rhythm genes play a crucial role in biological processes such as apoptosis, cell aging, DNA damage repair, and metastasis by rhythmically regulating gene expression and gene activity in the whole genome (Shafi and Knudsen, 2019; Crespo et al., 2021; Huang et al., 2023). More and more pieces of evidence show the importance of circadian rhythm genes in the diagnosis, treatment, and cancer outcomes (Angelousi et al., 2019; Qiu et al., 2019; Zhang et al., 2020; Sahar et al., 2022). This study found a correlation between PER2 expression and several core circadian rhythm genes, confirming the complex regulation mechanism network. If the rhythm expression of the PER2 gene oscillates and cannot be adjusted by the circadian rhythm regulation mechanism, it causes circadian rhythm disorders, which lead to many diseases, including liver cancer. Several studies found that the circadian rhythm plays an essential role in the occurrence and development of tumors, and “time therapy” has been recognized by many researchers and physicians (Zhou et al., 2021). Nevertheless, the mechanism of circadian rhythm genes, including PER2, in tumorigenesis and development is exploratory.

This study used bioinformatics analysis to determine the potential mechanisms of PER2 in liver cancer. We obtained a set of DEGs related to PER2 expression through single gene difference analysis and then analyzed their functions and pathways to understand their relationship to PER2. The DEGs related to PER2 expression were enriched in mitochondrial oxidative phosphorylation, transcription and translation, amino acid metabolism, and other related pathways. PER2 and other core circadian rhythm genes form a transcription-translation feedback loop; abnormal transcription and translation cause tumor proliferation, metastasis, and invasion. Experimental studies confirmed that the dysfunction of mitochondrial oxidative phosphorylation promotes the proliferation, metastasis, and invasion of primary liver cancer (Lee et al., 2021; Zhang et al., 2023). Branched-chain amino acid catabolism can break glutamine metabolism, maintaining HCC progression (Yang et al., 2022). These studies suggested that PER2 may be involved in the proliferation, metastasis, and invasion of HCC through mitochondrial oxidative phosphorylation, transcription and translation, and amino acid metabolism; nevertheless, we need further research and experiments to elucidate the mechanisms fully.

The role of immune cell infiltration in cancer development and progress has attracted substantial attention in recent years (Dai et al., 2022). This study found that PER2 expression in HCC showed a strongly negative correlation with pDC. There is pDC accumulation in blood, tumor tissue, and ascites in HCC patients, and high-density tumor infiltration of pDC is related to poor outcomes (Pang et al., 2021). Pang et al. found that IFNα secreted by pDC induced postoperative recurrence of HCC by promoting the recruitment of marrow-derived inhibitory cells (Pang et al., 2022). PER2 may play an essential role in regulating the tumor immune microenvironment. Nevertheless, the mechanism by which PER2 affects the immune microenvironment and tumor progression in HCC remains unclear, and further research is needed to clarify the biological effects of PER2 in HCC.

In recent years, the application of immune checkpoint inhibitors has become common and has also significantly improved HCC outcomes (Wang Z. et al., 2023). TP53 mutates in about half of human malignant tumors, including breast, colon, lung, liver, prostate, bladder, and skin malignant tumors (Marei et al., 2021; Wang H. et al., 2023). TP53 mutations inhibit anti-tumor immunity and reduce the efficacy of cancer immunotherapy (Wang H. et al., 2023; Marvalim et al., 2023). Therefore, we evaluated the relationship between PER2 expression level, immune checkpoint genes, and the TP53 gene. PER2 expression positively correlated with immune checkpoint genes and TP53 expression in HCC tissues. Interestingly, studies showed that PER2 binding prevents MDM2-mediated ubiquitination of tumor suppressor p53, regulating the p53 stability (Gotoh et al., 2016). These findings suggest that PER2 is involved in liver cancer and may be a potential target to improve immunotherapy, contribute to the research and development of new drugs, and provide a strategy and research direction for immunotherapy for HCC.

DNA methylation is an epigenetic mechanism. Previous studies showed that the change of DNA methylation pattern is critical in HCC and has potential value for diagnosis and outcome prediction (Long et al., 2019; Qu et al., 2020). Ma et al. found that hypermethylation of five CpG loci of the EXO1 gene was related to poor overall survival of HCC patients (Ma et al., 2022), and Yuan et al. found that hypermethylation of two CpG loci of the DDX1 gene was related to poor outcomes (Yuan et al., 2022). However, no study has shown the relationship between the methylation level of the circadian rhythm gene PER2 and HCC outcomes. Therefore, we conducted methylation analysis and evaluated the relationship between the methylation level of each CpG site of the PER2 gene and HCC outcomes. We identified eight CpG loci, among which the hypermethylation of six CpG loci, cg20070418, cg22879834, cg21315421, cg24831107, cg12308675, and cg06259818 were associated with poor outcomes. These findings suggest that the methylation status of PER2 is related to HCC outcomes. Nevertheless, we need *in vivo* and *in vitro* evidence to confirm the relationship between PER2 methylation and HCC outcomes.

Our study has some limitations. Most of our results were based on RNA sequencing data of HCC tissues from the TCGA database. However, we could not verify the analysis results of bioinformatics by basic experiments. Therefore, further *in vivo* and *in vitro* experiments are necessary to investigate the mechanisms of PER2 in HCC.

## 5 Conclusion

The circadian rhythm gene PER2 was expressed at low levels in HCC, and its expression level was correlated with nerve invasion, Child-Pugh grading, CNLC staging, immune cell infiltration, and immune checkpoint genes. PER2 expression was positively correlated with TP53, while its methylation status was related to outcomes. DEGs related to PER2 are enriched in mitochondrial oxidative phosphorylation, transcription and translation, amino acid metabolism, and other pathways. Therefore, PER2 may be a new prognostic and therapeutic marker for HCC. However, further research is needed to validate our findings.

## Data Availability

Publicly available datasets were analyzed in this study. This data can be found here: https://portal.gdc.cancer.gov/; https://biit.cs.ut.ee/methsurv/.
