# Supplementary figures and images for: Period2 is associated with immune cell infiltration and is a potential diagnostic and prognostic marker for hepatocellular carcinoma
Source: Front Mol Biosci. 2023 Nov 21;10:1264553. doi: 10.3389/fmolb.2023.1264553 (PMC10702766; doi:10.3389/fmolb.2023.1264553)

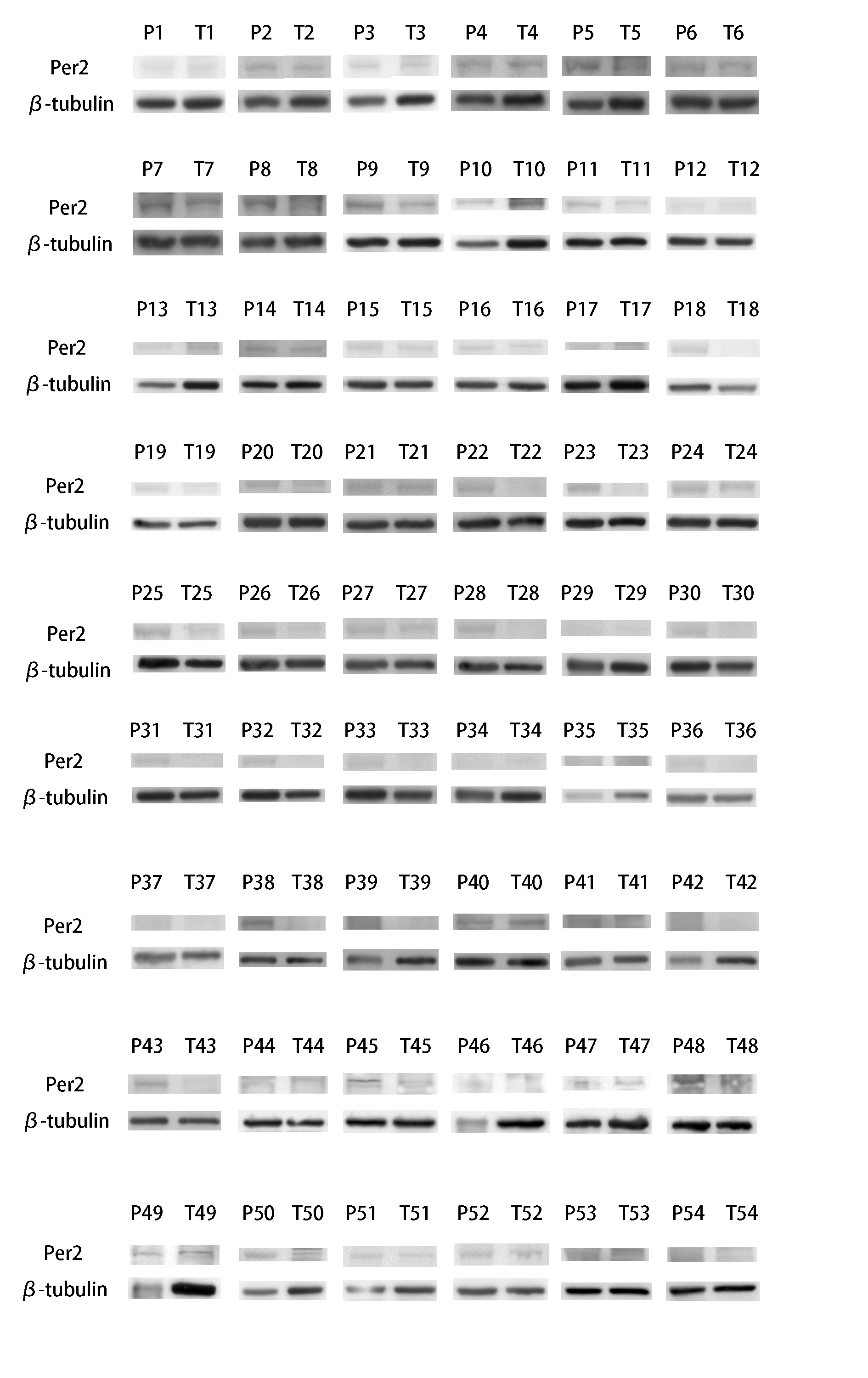

Supplement: Supplementary file 2 [file Image3.JPEG]

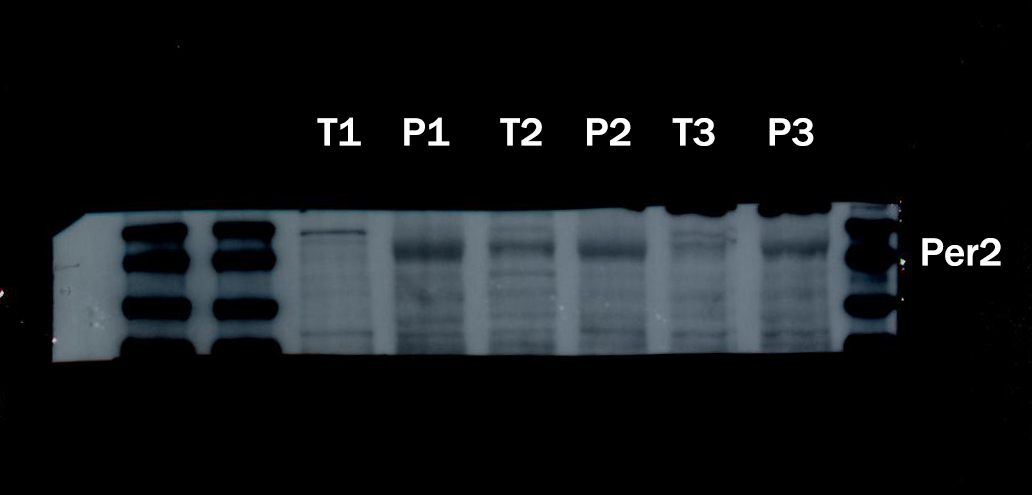

Supplement: Supplementary file 4 [file Image1.JPEG]

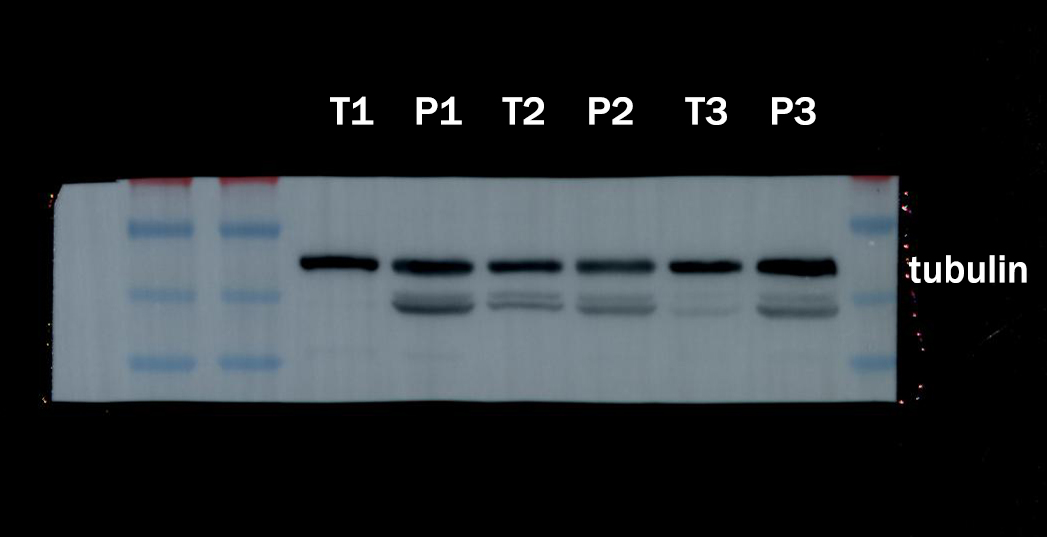

Supplement: Supplementary file 5 [file Image2.JPEG]
